# Computer-based surgical skill training: a systematic review

**DOI:** 10.1186/s12909-026-08830-7

**Published:** 2026-02-26

**Authors:** Lars Andreas Morsund, Shraddha Singh, Agastya Patel, Francesco Lancellotti, Thomas Satyadas

**Affiliations:** 1 Faculty of Medicine, Medical University of Gdańsk, Gdańsk, Poland; 2 Akershus University Hospital, Lørenskog, Sykehusveien 25, 1478 Norway; 3 1st Department of Cardiology, Medical University of Gdańsk, Gdańsk, Poland; 4 Cork University Hospital, Cork, Ireland; 5 First Doctoral School, Medical University of Gdańsk, Gdańsk, Poland; 6 Department of Hepato-Pancreatic and Biliary Surgery, Manchester Royal Infirmary, Manchester, UK

**Keywords:** Education, Surgical training, Computer-based learning, Remote teaching, Self-directed learner

## Abstract

**Background:**

Computer-based learning (CBL) has emerged as a transformative approach in surgical education, with remote teaching (RT) and self-directed virtual teaching (SDVT) offering scalable and, flexible alternatives to traditional face-to-face instruction. This systematic review aims to qualitatively analyse the current evidence on RT and SDVT for surgical skill training.

**Methods:**

In July 2023, we conducted a structured search of PubMed, Cochrane Library, Scopus, and Web of Science. To enable a clear evaluation of CBL, particularly by examining retained knowledge, we included only randomized controlled trials (RCT) and prospective cohort studies. Data extraction followed a pretested Excel form, and qualitative synthesis adhered to PRISMA principles.

**Results:**

Twenty seven studies (15 RCTs, 12 prospective cohorts) published between 2006 and 2023 met the inclusion criteria. Despite the heterogenicity of the included studies, key findings show that students completed RT achieved competency outcomes equivalent to traditional learning for basic suturing, knot-tying, and fundamental laparoscopic skills in most of the studies. SDVT matched traditional learning for basic tasks but underperformed in complex procedures. Hybrid models combining SDVT with real-time feedback enhanced learner autonomy, engagement and skill retention. Lastly, CBL modalities were well received by trainees, offering time- and cost advantages, as well as providing a teaching platform for low- and middle-income countries.

**Conclusions:**

CBL, through RT and SDVT, provides an effective adjunct to traditional surgical training, particularly for foundational technical skills. Integrating the flexibility of SDVT with synchronous feedback during RT can optimize psychomotor learning while maintaining instructional oversight. Future research should standardize outcome measures, assess long-term skill retention, and explore implementation in resource-limited settings.

## Introduction

The recent COVID-19 pandemic disrupted education globally, affecting more than 94% of students worldwide [1]. In response to public health mandates issued by the World Health Organization [2], educators rapidly adopted alternative instructional modalities. Despite the rapid expansion of computer-based instructional modalities in surgical education, the effectiveness of synchronous versus asynchronous virtual teaching for psychomotor skill acquisition remains unclear. A structured synthesis of these approaches is needed to guide evidence-based curriculum design.

This systematic review qualitatively synthesizes the current evidence on camera-based and video-based instructional approaches for surgical skills training. Specifically, it examines Self-Directed Virtual Teaching (SDVT) and Remote Teaching (RT), as compared to Traditional Learning (TL), which typically involves face-to-face instruction in classroom or simulation center settings.

SDVT refers to the independent acquisition and assimilation of information without direct instructor involvement [3]. It commonly involves pre-recorded videos, slide presentations, and other asynchronous materials. On the other hand, RT includes real-time, synchronous teaching through video conferencing platforms such as Zoom™, Google Meet™ or Microsoft Teams™, where learners perform tasks under direct online supervision.

This review is focused exclusively on technical psychomotor skills, including suturing, knot-tying, and laparoscopic maneuvers. Through a structured and protocol-driven literature search, we aim to provide a comprehensive narrative synthesis of effectiveness of these computer-based learning modalities and to offer recommendations for their integration into modern curricula.

## Methods and materials

We conducted a systematic review, guided by the Preferred Reporting Items for Systematic Reviews and Meta-Analyses (PRISMA) framework [4]. Due to the study heterogeneity and varied outcome measures, we synthesized results thematically rather than quantitatively. A predefined protocol outlining the research question, search strategy, and eligibility criteria was developed a priori. Although the protocol was not prospectively registered, the review methodology followed PRISMA guidelines; a detailed copy is available from the corresponding author upon reasonable request.

### Literature search

**Fig. 1:**
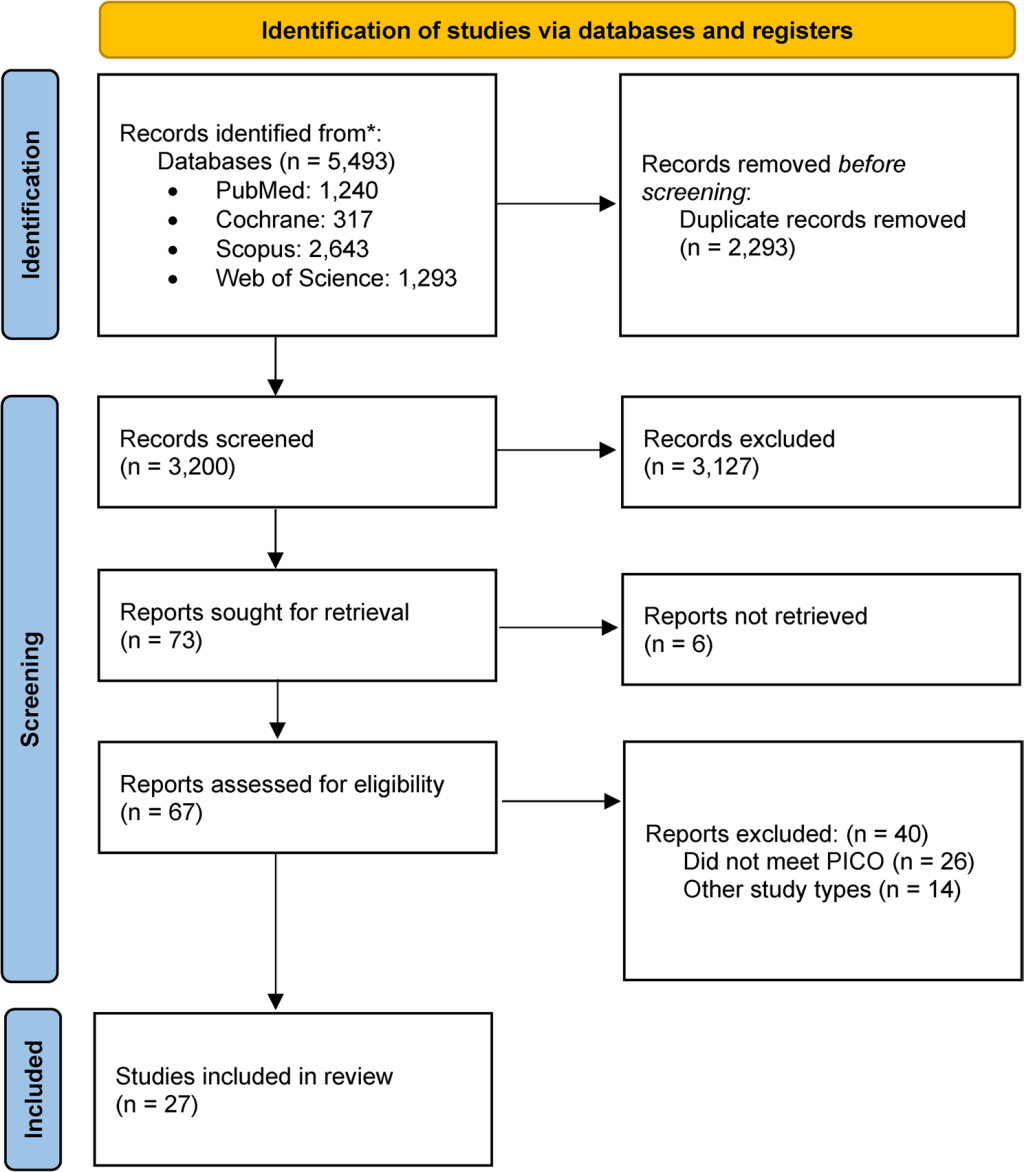
PRISMA flowchart illustrating the inclusion of eligible articles. *From*: Page MJ, McKenzie JE, Bossuyt PM, Boutron I, Hoffmann TC, Mulrow CD, et al. The PRISMA 2020 statement: an updated guideline for reporting systematic reviews. BMJ 2021;372:n71. doi: 10.1136/bmj.n71

The PubMed, Cochrane Library, Scopus and Web of Science databases were used to find suitable articles. Language filters were not applied during the search. Backward chaining was performed to find eligible full-text studies. On the 18th of July 2023, the search was performed with the following search strategy: (“remote” OR “computer-based” OR “online” OR “video-based” OR “tele simulation” OR “e-learning”) AND (“surgical skill*” OR “laparoscopic skill*” OR “suturing” OR “knot-tying” OR “knotting” OR “surgical training”). The preliminary search yielded 5,493 articles. A total of 3,200 duplicates were removed. Following title and abstract screening, the full-texts of 73 articles were reviewed, and 27 articles were ultimately included [5–31]. Two authors (LAM and SS) screened the abstracts and identified articles eligible for this review, but any discrepancies were resolved through discussion with a third author (AP). The PRISMA flowchart is illustrated in Fig. 1.

### Eligibility criteria

This review was based on a participant, intervention, comparison and outcomes (PICO) framework to identify and phrase the research questions. The inclusion criteria were as follows: Participants - medical students or surgical residents receiving practical surgical education; Intervention - SDVT and/or RT involving basic or advanced surgical skills; Comparison - TL modalities; Outcomes - evaluation of the interventions using objective and subjective measures, such as self-assessment and satisfaction surveys [32]. Objective scoring systems included the Objective Structured Assessment of Technical Skills (OSATS) [33], Global Rating Scale (GRS) [33], Global Operative Assessment of Laparoscopic Skills (GOALS) [34], Operative Performance Rating System (OPRS) [35], Suturing and knot-Tying Training and Testing (SUTT1) [36], and multiple-choice questions. Additionally, preintervention testing (PRT), postintervention testing (POT) and retention test (RET) results were used in the majority of the studies to assess learning and retention of skills.

To enable a clear evaluation of intervention effects, particularly by examining retained knowledge post-intervention, we included only randomized controlled trials (RCTs) and prospective cohort studies. We grouped procedural tasks into basic and advanced skill domains to reflect escalating technical complexity and cognitive load. Basic skills include tasks such as knot-tying, simple interrupted suturing, instrument handling, and wound closure. These form the psychomotor foundation for novices and are typically taught in early surgical curricula. Objective measures (e.g., knot count, time to completion) provide straightforward performance metrics. Advanced procedures requiring deeper visuospatial integration and fine motor control, including laparoscopic suturing, microsuturing, bowel anastomosis, and intracorporeal knot-tying. These tasks involve dual-hand coordination, scope navigation, and complex decision-making under simulated operative conditions. Assessment tools such as GOALS and advanced OSATS scales capture the nuanced quality, and efficiency demands of these higher-level skills. This division fits with established surgical education frameworks and allows for evaluation of how SDVT and RT performs across varying levels of technical challenges.

Immersive virtual reality and artificial intelligence-assisted platforms were excluded. These modalities engage broader constructs, beyond direct camera-based skills, and would introduce great heterogeneity in both instructional design and outcome assessment, limiting comparability with SDVT and RT. The exclusion criteria were articles for which the full text was not available, those not written in English, those that did not meet our PICO framework.

A pretested data extraction form was created using Microsoft Excel™ and the following data were extracted from each of the eligible studies: first author, year, trial site, study design, participants, training task, groupings, number of participants, intervention/control, outcomes measured, and outcomes. Disagreements between the authors were resolved through discussion.

Risk-of-bias assessment was planned for randomized controlled trials. Non-randomized studies were considered too methodologically heterogeneous for formal appraisal and were therefore narratively evaluated.

### Outcomes

The primary outcome was objective quality assessments of trainee performance, while the secondary outcome included subjective data collected from self-assessments and satisfaction surveys. In total, 25 of the 27 studies reported objective outcomes. The key factors evaluated in the tests were the time taken to perform a predefined task, the number of knots or sutures tied and the number of hand movements in a set time window. The self-assessment tests included surveys in which participants ranked their performance and level of satisfaction with the modality.

## Results

Twenty-seven articles met the predefined eligibility criteria and were included in this review. Twelve articles focused on RT [5, 6, 9, 15, 21, 24, 27, 28, 30, 31], of which two studies compared education in basic surgical skills through RT, SDVT and TL [12, 29]. Fifteen of them were SDVT [7, 8, 10, 11, 13, 14, 16–20, 22, 23, 25, 26].

### Risk of bias in included studies

Risk of bias was assessed independently by two reviewers using the Cochrane Risk of Bias 2 (RoB 2) tool for randomized controlled trials. The majority of randomized trials demonstrated a low overall risk of bias, while several studies raised some concerns, primarily related to the randomization process. No randomized trials were classified as having a high risk of bias. Due to substantial methodological heterogeneity and the predominantly descriptive design of the prospective non-randomized studies, a formal risk-of-bias assessment was not performed for these studies; therefore, their findings should be interpreted with appropriate caution.

### Remote teaching 

This review analysed 12 studies about RT of basic (n = 7) and advanced (n = 5) surgical skill (Table 1). RT demonstrated comparable effectiveness to TL in most studies. Four trails reported no difference in objective scores basic surgical skill training between the groups [6, 24, 27, 30], suggesting RT achieves equivalent competency outcomes. One study reported only subjective results showing high level of student satisfaction with the RT-modality [5]. Additionally, two studies compared all three modalities [12, 29]. One study found no significant difference among modalities, though all participants improved. The other study highlighted RT and TL outperformed SDVT, with subjective feedback favouring RT over TL. Evidence for RTs effectiveness in advanced laparoscopic training was more variable. Three cohort studies showed significant improvement in laparoscopic skills improvement [21, 28, 31], while RCT findings were mixed. One trail showed no difference compared to TL [9], whereas another identified RT as superior in performance outcomes [15].

**Table 1:** Remote teaching for basic and advanced surgical skills

Author and Country	Study design	Task	Groupings	Participants	Interventions
RT of Basic Surgical Skills					
Alameddine et al. 2018, USA [6]	RCT	Continuous running suture	TL	8	PRT → Practice → Break (10 min) → POT
			RT	8	PRT (recorded) → Coaching by examining video of PRT (10 min) → POT
Co et al. 2020, Hong Kong [5]	PS	Instrument handling, knot-tying and suturing	RT	30	Live, online demonstration of surgical knot tying → Practicing with feedback → POT
Nathan et al. 2021, United Kingdom [12]	RCT	Knot-tying, 3 interrupted sutures	TL	24	PRT → Instructional video (6 min) → Practice (90 min) with instructor-to-student ratio 1:4 → POT
			RT	24	PRT → Instructional video → Practice (via weConnect™) with feedback → POT
			SDVT	24	PRT → Instructional video → Practice (via Zoom™) while access to the video → POT
Feeley et al. 2022, Ireland [30]	RCT	Simple interrupted sutures	TL	13	PRT→ In-person practice classes → POT
			RT	10	PRT → Livestream of the TL group → POT
Pinter et al. 2022, Hungary [27]	RCT	Knot-tying and suturing	TL	30	PRT → 5 days with 4 h of classes. Practicing with a SkillBox™ after watching tutorial videos and demonstrations in the classroom → POT
			RT	30	PRT → 5 days with 4 h of classes. Practicing with a SkillBox™ at home after watching tutorial videos and receiving feedback from the instructors → POT and Likert survey
Zaghal et al. 2022, Lebanon [24]	RCT	Interrupted sutures	TL	59	Watch instruction video two times followed by a demonstration → Practice with possible feedback → POT
			RT	59	Meeting on WebEx™ → Watch instruction video two times followed by a demonstration by the instructor → Practice with possible feedback → POT
Fehervari et al. 2022, United Kingdom [29]	PS	Scrubbing and theatre safety, surgical instruments, suturing and knot tying	TL	20	PRT (questionnaire) → Face-to-face lecture teaching → POT (questionnaire)
			RT	64	PRT (questionnaire) → Practice in live teaching sessions with online discussion forums → POT (questionnaire)
			SDVT	489	PRT (questionnaire) → participants had the possibility to also practice in breakout-room (1:5, instructor to students’ ratio) with online discussion forums → POT (questionnaire)
RT advanced surgical skills					
Singh et al. 2015, England [15]	RCT	Laparoscopic cholecystectomies	TL	10	Five task attempts with online lectures (30 min) after each attempt → POT
			RT	10	Five task attempts with expert feedback after each procedure, with video-recordings → POT
Bilgic et al. 2022, Canada [21]	PS	Laparoscopic needle suturing	RT	6	PRT → Control period after 1 month → Six to eight practice sessions via Skype™ FFT was performed → POT
Akdemir et al. 2022, Turkey [31]	PS	Laparoscopic C-loop square knot	RT	17	Box trainer was sent to participants → Instruction video → Meeting (Zoom™) with instructor-to-trainee ratio 1:4 → PRT → Practice with feedback → POT
Lowry et al. 2022, Canada [9]	RCT	FLS	TL	10	Demonstration → PRT → In-person practice (2 h) → POT → RET after 4 weeks
			RT	10	Demonstration → PRT → Practice (2 h) via HelpLightning™ → POT → RET
			Control	10	Demonstration→ PRT→ Self-practice (2 h) without feedback → POT → RET
Ramadan et al. 2023, Canada [28]	PS	FLS	RT	11	PRT (survey) → 60 min session per week for three weeks on Zoom™ with break-out rooms → POT (official FLS exam, survey and Likert scale)

*Abbreviations*: *CBL *Computer-based learning, *FFT *Formative feedback tools, *FLS * Fundamental laparoscopic surgery, *GOALS *Global Operative Assessment of Laparoscopic Skills, *GRS *Global rating scale, *NR * Not reported, *OPRS *Operative Performance Rating System, *OSATS *Objective Structured Assessment of Technical Skills, *PS *Prospective cohort study, *RCT *Randomized control trail, *RT * Remote teaching, *SDVT *Self-directed virtual teaching, *TL *Traditional teaching

### Self-directed virtual teaching

A total of 15 SDVT studies analysed basic (*n* = 11) and advanced (*n* = 4) surgical skills (Table 2) [7, 8, 10, 11, 13, 14, 16–20, 22, 23, 25, 26]. These studies yielded mixed results, with some studies showing that SDVT is as effective as TL, while others concluded with TL being superior.

**Table 2:** Self-directed virtual teaching for basic surgical skills

Author and Country	Study design	Task	Groupings	Participants	Interventions
SDVT of Basic Surgical Skills					
Brandt et al. 2006, Canada [11]	RCT	Knot-tying	TL	30	Mental Rotations Test → Presentation with verbal instructions (10 min) → Practice (10 min) → POT
			SDVT	30	Mental Rotations Test → Presentation with written instructions (10 min) → Practice (10 min) → POT
Xeroulis et al. 2007, Canada [7]	RCT	Knot-tying and suturing	TL	15	PRT → Instructional video → Practice → POT → RET (after 1 month)
			SDVT	15	PRT → Instructional video → Practice with option of interaction with video demonstration and narration from an expert → POT → RET
			Concurrent feedback	15	PRT → Instructional video → Practice with feedback → POT → RET
			Summary feedback	15	PRT → Instructional video → Practice with feedback after each trail → POT → RET
Scott et al. 2007, USA [22]	PS	Instrument handling, knot-tying and suturing	SDVT	4	Video tutorial (43 min) → PRT → Self-trained to proficiency → POT
Nousiainen et al. 2008, Canada [8]	PS	Suturing	SDVT: Group 1	8	Watched instruction video only once → POT
			SDVT: Group 2	8	Access to video during practice attempts → POT
			SDVT: Group 3	8	Access to video and feedback during practice → POT
Wright et al. 2012, USA [20]	PS	Instrument handling, knot-tying, basic wound closure	SDVT	9	Instruction video → PRT → Practice with online material → POT
Chien et al. 2015, USA [10]	RCT	Laceration repair	TL	20	Lecture with demonstration (20 min) → Practice (100 min) with feedback → POT → RET
			SDVT	20	Video of the TL lecture session → Practice (100 min) without feedback → POT → RET
Lwin et al. 2018, Myanmar [13]	RCT	Knot-tying tasks, Suturing tasks	TL	25	Video demonstration → PRT → Practice with instructions and feedback (60 min) → POT
			SDVT	25	Video demonstration → PRT → DVD and practice (60 min) → POT and survey
Yuda et al. 2021, Indonesia [16]	PS	Knot-tying	TL	39	PRT → Online Class via Zoom™ → POT
			SDVT	50	Tutorial video (3.5 min) → PRT →Zoom™ class → POT
McGann et al. 2021, USA [23]	PS	Instrument handling Knot-tying tasks, Suturing tasks	SDVT	86	PRT → Three self-paced e-Learning module with evaluation after each module → Direct feedback (30 min) → POT (survey also for faculty members)
Kumins et al. 2021, USA [19]	PS	Instrument handling, Knot-tying tasks, Suturing tasks	SDVT	221	PRT → 12 stepwise tutorial videos (3–15 min) with surveys → Practice→ POT
Nematian et al. 2023, Iran [26]	RCT	Interrupted sutures	SDVT-Noninteractive	53	PRT → Noninteractive access to narrated video → POT → RET (after 1 month)
			SDVT-Interactive	53	PRT → Interactive access to narrated video while practicing on a simulator → POT → RET
			TL	53	PRT → Simulator practice with feedback. Student to instructor ratio 7:1 → POT → RET
SDVT advanced skills					
Rowse et al. 2015, USA[17]	PS	Hand-sewn small bowel anastomosis	TL: PGY-5	7	PRT → No access to the instructional video; Not aware of the ongoing study
			SDVT: PGY-1	25	PRT → Instructional video (13 min) → Practice → POT → RET (after 4 months)
Sakamoto et al. 2017, Japan [18]	RCT	Microsurgery knot-tying	TL	45	Instructional video (10 min) → Practice (40 min) → POT
			SDVT	41	Practice (60 min) with feedback → POT
Sloth et al. 2021, Denmark [14]	RCT	FLS	TL	24	PRT → Two training days (7 h per day. Instructor-to-trainee ratio 1:3) → POT → RET (after six months)
			SDVT	22	PRT → Practice at home for six weeks with online video instructions → POT → RET
Romero et al. 2023, Germany [25]	RCT	C-loop square knot	TL	27	Pyeton’s Four-Step Approach with student-teacher ratio 1:1. → POT
			SDVT	28	Access to instructional videos while practicing → POT

*Abbreviations*: *FLS *Fundamental laparoscopic surgery, *GRS *Global rating scale, *MCR *Multiple choice questions, *NR *Not reported, *OSATS *Objective Structured Assessment of Technical Skills, *PGY * post-graduation year, *PQS *Product quality score, *PS *Prospective cohort study, *RCT *Randomized control trail, *RT *Remote teaching, *SDVT *Self-directed virtual teaching, *SUTT1 *Suturing and knot-tying training and testing, *TL *Traditional teaching

The majority of the SDVT studies targeted basic technical skills such as instrument handling, suturing and knot-tying. Three RCTs found SDVT comparable to TL [7, 11, 13], especially when instructor-led feedback was integrated. One RCT [26], however, favoured TL to be superior to SDVT. Additionally, six prospective cohort studies [8, 16, 19, 20, 22, 23] showed significant improvement after the participants had completed the training session. Evidence for SDVT in advanced surgical training was more nuanced. Two RCTs reported performance outcomes comparable to TL [14, 18], while another trail favoured TL [25], highlighting the limitations of this modality when training for more complex procedures. One prospective cohort study found no significant difference in RET scores between trainees with different backgrounds, suggesting SDVT may support diverse learner profiles when appropriately framed [17].

## Discussion

CBL modalities represent a promising adjunct to traditional methods in surgical education, particularly in establishing foundational psychomotor skills [6, 13, 20]. This review suggests that CBL, specifically SDVT and RT, may offer comparable outcomes to TL under certain conditions. Among the included studies, RT emerged as a potentially more effective modality than SDVT in several settings, largely due to synchronous feedback from instructors [6, 37, 38]. However, SDVT also demonstrated utility in developing early-stage technical competencies such as suturing and instrumental handling, which are essential for preparing medical students for surgical rotation [10, 20].

A hybrid instructional design combining SDVT and RT could maximize the benefits of each modality [5, 14, 23]. Some studies proposed that such integration may improve learner autonomy while maintaining instrumental oversight. It is also notable that one included study reported a cost reduction of 13.2% per student when using CBL as compared to TL [27]. While this observation is promising, cost-efficiency was not consistently reported across studies, and therefore this benefit should be interpreted cautiously.

Time efficiency was mentioned in selected studies, which highlighted reductions in supervised contact hours and greater flexibility for both students and educators [6, 9, 12]. However, this review did not perform a meta-analysis or pooled time data; thus, conclusion on efficiency remain qualitative and context dependent. Similarly, although CBL may offer scalability and broader geographic reach, claims regarding its potential to address shortage of qualified surgical educators remain speculative [13, 28, 39, 40]. None of the included studies quantitively assessed this outcome.

Several challenges and limitations of CBL delivery were consistently reported. Studies conducted during the COVID-19 pandemic reported a reduced sense of learner-instructor connection and lower peer interaction in remote environments [41]. Lack of real-time feedback, limited motivation, and difficulty in self-assessment were additional concerns on SDVT formats [42, 43].

Resource availability like access to surgical kits, stable internet and appropriate devices, was another frequently cited barrier to successful implementation [9, 12, 13, 17, 21, 31].

To enhance the learner engagement in RT environment, tools such as breakout rooms and audience-response platforms (e.g., Mentimeter™) can facilitate interaction, provide formative assessment, and mimic elements of in-person education, which allows students to participate in polls and quizzes, thus increasing the engagement and interactivity of RT [44].

The concept of ‘Mastery learning’, wherein students take ownership of their own educational progression through individualized pacing and repeated practice, resonates strongly with pedagogical framework of SDVT [43]. Rather than emphasizing final assessments alone, this approach promotes continual learning through milestone-based evaluations. To support such models, the educational environment must be adaptable, offering structured opportunities for learners to engage in iterative practice and receive meaningful feedback.

Incorporating objective, structured scoring systems into self-directed curricula can enhance learner motivation and allow for progress monitoring [45]. In this context, self-assessment while subject to potential bias remains a valuable tool for reinforcing self-awareness and skill tracking [7]. For instance, students with lower self-efficacy or those who rely more on external validation (*dependent* learners) may benefit from the use of engaging visual aids or progressive dashboards, such as infographics. Educators should be sensitive to the spectrum recognize the gap between their teaching material and learners’ readiness for independent study and design materials that facilitate the transition from dependency to autonomy in learning.

### Limitations

This systematic review offers a comprehensive synthesis of computer-based modalities applied to psychomotor enhancement of surgical skills. However, this review has several limitations. Considerable heterogeneity existed across study populations, training tasks, instructional designs, and outcome measures, which precluded quantitative synthesis. Additionally, the absence of formal risk-of-bias assessment for non-randomized studies may limit the strength of conclusions derived from these data. Finally, variation in assessment tools highlights the need for standardized outcome reporting in surgical education research. several limitations must be acknowledged.

Another potential limitation of the current review is that the included studies span multiple countries, primarily high-income countries therefore, they may not be representative of low- or middle-income countries.

## Conclusion

Despite these limitations, this review represents an extensive qualitative synthesis of computer-based surgical skill training. The collective evidence supports the integration of RT and SDVT as meaningful adjuncts to traditional education, particularly when paired with expert feedback. These modalities offer scalable and cost-effective alternatives, with potential relevance in designing programs for emerging areas such as artificial intelligence integrated learning and robotic surgery.

However, it is essential to emphasize that while CBL can enhance technical skill acquisition, it cannot substitute for clinical experience, which remains irreplaceable in the development of competent, safe, and adaptable surgeons.

## Data Availability

The datasets used and/or analyzed during the current study are available from the corresponding author upon reasonable request.
